# Public and Patient Involvement in Artificial Intelligence and Big Data Healthcare Research: An Exploration of Issues and Challenges Within the AI‐Multiply Project

**DOI:** 10.1111/hex.70490

**Published:** 2025-11-14

**Authors:** Alexandra Thompson, Victoria Bartle, Elizabeth A. Remfry, Duncan J. Reynolds, Michael R. Barnes, Nick J. Reynolds, Barbara Hanratty

**Affiliations:** ^1^ Population Health Sciences Institute Newcastle University Newcastle Upon Tyne UK; ^2^ The AI‐Multiply Consortium; ^3^ AI‐Multiply PPIE Group; ^4^ William Harvey Research Institute Queen Mary University of London London UK; ^5^ Wolfson Institute of Population Health Queen Mary University of London London UK; ^6^ Institute of Translational and Clinical Medicine Newcastle University Newcastle Upon Tyne UK

**Keywords:** artificial intelligence, big data, public involvement

## Abstract

**Background:**

Public and patient involvement and engagement (PPIE) is intended to shape research priorities and improve relevance and impact. However, implementing PPIE in complex fields such as artificial intelligence (AI) and big data health research presents specific challenges. This study explores the issues and barriers to meaningful PPIE using the AI‐Multiply project as a case example.

**Methods:**

AI‐Multiply is a large, interdisciplinary UK‐based research project using AI and routine health data to investigate trajectories of multiple long‐term conditions and polypharmacy. PPIE was embedded across all five work packages. We used a mixed‐methods approach, drawing on CUBE framework surveys, PPIE feedback forms and impact logs to evaluate involvement. Data were analysed thematically using a ‘follow‐a‐thread’ approach to identify key issues across sources.

**Results:**

Three themes were identified: (1) **differing priorities**—public contributors prioritised person‐centred outcomes, while researchers focused on data‐driven healthcare metrics, often constrained by data availability; (2) **movement on both sides**—both researchers and contributors expressed early apprehension, but mutual trust and integration developed over time; and (3) **the importance of established guidance**—many issues raised echoed longstanding PPIE guidance on clarity, feedback and facilitation.

**Conclusion:**

While AI and data‐specific challenges exist, many PPIE issues in this context relate to applying existing good practice in complex projects. Strong PPIE leadership, early expectation‐setting and consistent facilitation are critical for success. Findings will inform the development of practical tools to support involvement in data‐driven research.

**Patient or Public Contribution:**

Public contributors with lived experience of multiple long‐term conditions contributed to the interpretation of data and co‐authored this manuscript.

## 1 Introduction

Public and patient involvement and engagement (PPIE) in research is defined by the UK National Institute for Health and Care Research (NIHR) as research carried out *with, by* or *in partnership with* members of the public, rather than *to, about* or *for* them [1]. While its growth has often been driven by funder expectations, PPIE has genuine potential to shape priorities and enhance the relevance and impact of research [2, 3, 4]. The UK Standards for Public Involvement [5] outline the principles of good practice, including the use of plain language, inclusivity and timely feedback. A range of methods also exists for evaluating the process and impact of involvement [6]. However, existing guidance often lacks practical procedures for implementation, particularly in more complex domains.

This paper evaluates PPIE within a research programme exploring artificial intelligence (AI) in the context of multiple long‐term conditions (MLTCs) to highlight some of the challenges and lessons learned when applying PPIE in cross‐disciplinary research.

### 1.1 Background

There has been a rapid growth in AI and ‘big data’ research over the past decade, alongside increasing interest in their healthcare applications [7]. AI is generally defined as computer systems capable of simulating human learning, problem solving or creativity [8]. These systems rely on large datasets, often electronic health records, for training and optimisation [9].

AI and big data health research raise ethical, social and technical challenges that make a strong case for PPIE [10, 11]. For example, the underrepresentation of marginalised groups in health data may produce algorithmic bias and reinforce health inequalities [12, 13, 14, 15]. The training and decision‐making processes of AI systems are rarely transparent, making human scrutiny vital [16]. Concerns also exist around loss of clinician input, the potential dehumanisation of healthcare [17] and risks to data privacy.

Media portrayals of AI have further fuelled mistrust and misinformation [18]. Meaningful public involvement has been suggested as a mechanism to address these concerns and ensure AI delivers real patient benefit [12]. Some argue that the public should be active stakeholders in AI governance, not passive recipients [19], and that involvement can help ensure public service values remain central to technological development [20].

Even in more traditional research settings, PPIE faces known challenges, such as limited researcher confidence or skills, and tension between public and expert perspectives [21, 22]. These tensions may intensify when public partners are given greater agency. While this may amplify the public voice, it can also generate friction if researchers' goals are shaped by a diverse range of views and experiences [23].

In AI and big data health research, these difficulties are compounded by the abstract nature of the field, public unfamiliarity, low baseline knowledge, trust issues and broader social attitudes [13, 24]. Supporting active public involvement in this context may require new methods and more time, for example, to explain data science approaches, data protection and how AI can work with available data. Understanding the challenges of doing this well is key to building effective models for future research.

Despite the wide availability of PPIE guidance, over 500 toolkits are currently in circulation [25], most focus on general principles rather than practical implementation [26]. None are tailored specifically to AI or big data research. Some include relevant elements, such as building public trust in technology, but overlook challenges around representation, baseline knowledge, digital literacy or inclusive communication [12]. Participatory approaches in AI development exist, but often focus on high‐risk regulation [27] or have a commercial orientation [28]. These rarely translate well to health and care contexts.

### 1.2 A Case Study—AI‐Multiply

AI‐Multiply is a large NIHR‐funded, interdisciplinary project exploring how AI and established analytical approaches can improve care for people with MLTCs and polypharmacy. It involves five work packages focused on predicting and understanding disease trajectories (see Box 1). PPIE was embedded from the outset, supported by two public contributor groups: one UK‐wide (initially 30 members) and a second based in London, facilitated by the charity Social Action for Health.

BOX 1 **Work packages within AI‐Multiply.**

**WP1—Preparing data for analysis**

This work package focuses on identifying and linking electronic health record (EHR) data from regional and national sources to prepare for analysis.

*PPIE partners advised on data usability, gaps in lived experience and relevance to patient priorities*.

**WP2—AI for multiple long‐term condition (MLTC) and polypharmacy clusters, changes over time**

Artificial intelligence techniques are used to identify clusters of conditions and medications, and how they change over time in relation to social and individual factors.

*Contributors helped shape outcome priorities*.

**WP3—Interpretation and clinical decision‐making**

This work package explores how MLTCs and treatments relate to specific groups of conditions (e.g., mental health and inflammatory disorders) and patient characteristics.

*PPIE contributors informed research focus and supported interpretation of patterns that align with everyday experiences*.

**WP4—Interdisciplinary entanglements: emergence of ideas, consensus and technologies in scientific practice**

Ethnographic methods are used to understand how collaboration happens across disciplines in AI and healthcare research.

*Contributors co‐developed the project's mission statement and supported team reflections on inclusive working and shared values*.

**WP5—Health and social care outcomes, translation into practice**

This work package focuses on how findings can inform practice, including emulated clinical trials using EHR data.

*PPIE helped develop trial questions and highlighted the need to measure outcomes that reflect patient concerns*.

**Cross‐cutting Theme—Towards an intersectional understanding of inequalities**

All work packages contribute to analysis of MLTC patterns based on structural and social factors (e.g., ethnicity, gender and deprivation).

*PPIE advised on how inequalities were defined, interpreted and addressed across the research*.

This project presented an unusual PPIE opportunity due to the size of the contributor group, their geographical spread and the diversity of research packages involved (see Section Methods, 2). These features created a valuable setting to evaluate PPIE practice in big data and AI research.

In this article, we aim to evaluate the implementation of PPIE within AI‐Multiply and explore the distinct issues and challenges it presented. Our objectives were to identify areas for improvement, understand how AI and big data affect PPIE and offer practical recommendations for future implementation in this domain.

## 2 Methods

### 2.1 PPIE Group Formation

AI‐Multiply involved two PPIE groups. The first was coordinated by the PPIE management team in Newcastle, recruited through local networks including VOICE UK [29] and Cumbria, Northumberland, Tyne and Wear NHS Foundation Trust (CNTW) [30] and began with 30 UK‐wide members (some attrition occurred over time. Contributors came from diverse educational and professional backgrounds; some were familiar with research and technical content, others less so. Due to growing organisational demands, the PPIE management team was expanded to include six public contributors and one researcher.

The second group, formed by Social Action for Health in East London, is a charity focused on engaging underserved populations. This charity works with people affected by health inequalities, including individuals with limited English or from minoritised ethnic backgrounds. They recruited 22 participants from their community projects supporting people with MLTCs. Sub‐groups were later formed to support ongoing research. *This paper evaluates the ‘Newcastle’ group only*.

### 2.2 PPIE Format and Operation

Rather than following a fixed plan, PPIE activities were developed flexibly to meet researcher needs across work packages and adapt to contributor feedback. PPIE was comprehensively integrated across the project through proactive identification of areas for lived experience input and actively creation of opportunities for involvement. In this way, PPIE was embedded in full team and steering meetings. This integration also placed PPIE contributors within whiteboard and data engineering meetings, spaces traditionally perceived as too technical for public input.

As many early career researchers (ECRs) had limited PPIE experience, dedicated fortnightly ECR‐PPIE sessions were established midway through the project. These informal, 1‐h meetings gave ECRs opportunities to present technical ideas and receive feedback. Participation grew over time despite early hesitation [31].

Full‐group online workshops were held at the start to build baseline knowledge on AI, data science and the role of PPIE. Later, each work package had at least one dedicated session. All contributors were invited to most sessions, though some were split into parallel or sequential series. Sessions typically lasted 1 h and combined presentations with guided discussion.

### 2.3 PPIE Facilitation and Evaluation Methods

We drew on NIHR's Co‐Production Guidance [32] to conceptualise PPIE within AI‐Multiply, emphasising power‐sharing, valuing all perspectives and mutual learning, vital for a technically complex, interdisciplinary study. The UK Standards for Public Involvement [5] informed our practice. For evaluation, we used three complementary tools endorsed by People in Health, West of England (PHWE) [6] and NIHR:

- **CUBE surveys**: Public contributors' perspectives.
- **Impact logs**: Researcher and team reflections.
- **Feedback forms**: Session‐level evaluations.

These tools enabled triangulated, real‐world evaluation of PPIE activity across work packages.

While most evaluation participants were public contributors, some ECRs and researchers also provided input via feedback forms and logs. However, systematic collection of researcher perspectives is planned for a future phase.

1. Planning and reviewing PPIE: The Public Involvement Impact Assessment Framework (PiiAF)

We initially used PiiAF [33] to help researchers design involvement activities and plan for impact evaluation. Developed by researchers and NIHR contributors, PiiAF supports structured planning. However, time constraints and perceived burden led to an adapted approach, which still allowed for planning of PPIE sessions and documenting impacts:

Before sessions, researchers outlined their goals for PPIE; afterwards, the PPIE team followed up to log actions taken based on contributor input. These were recorded in the impact log.

### 2.4 Recording PPIE Outcomes: The Impact Log

Adapted from PHWE guidance, the impact log was used to track PPIE activity and observed effects. Maintained by the PPIE lead (and at times, researchers or PPIE team members), it documented:

- Work package and activity info (title, date and time)
- Attendance and detailed discussion notes
- Impacts of involvement (including immediate outcomes)
- Assessment strategies for outcomes
- Reflections and learning to improve future sessions

2. Eliciting PPIE contributor views on PPIE process, quality and impact: The CUBE framework

We used the CUBE framework [34] to evaluate PPIE quality and process from the public contributors' perspectives. It was well‐suited to this context, as it captures power dynamics, communication and openness to influence, crucial in interdisciplinary and technically complex work.

The framework assesses four dimensions: **strength of voice, ways to be involved, focus of research concerns and willingness to change**. For each PPIE session, an online survey was shared. Contributors rated their perceptions of involvement on a 1–10 scale and could add free‐text context. An additional interactive exercise was conducted at an in‐person event. Free‐text responses generated tailored recommendations for researchers.

## 3 Evaluation of Data Sources

For this evaluation, we systematically analysed CUBE responses, impact logs and feedback forms originally created as part of routine monitoring (see Table 1).

**Table 1:** Summary of evaluation of data sources.

Data source	Participant type	Purpose	Collection method	Linked work packages
CUBE framework surveys	Public contributors	Evaluate public contributor experience across 4 involvement dimensions	Online survey and interactive session	All WPs
Impact logs	PPIE team, researchers and public contributors	Document PPIE‐related changes, reflections and process insights	Narrative logs maintained by the team	All WP PPIE sessions plus data engineering and whiteboard meetings
Feedback forms	Public contributors and researchers	Capture immediate feedback after PPIE sessions	Structured feedback forms	ECR‐linked sessions across all WPs
ECR session reflections	Public contributors and ECRs	Assess mutual learning and comfort with technical material	Open‐text responses post‐session	All WPs
Researcher reflections in logs	Researchers	Reflect on integration and attitudes to PPIE over time	Narrative entries in logs	Various

*Note:* This table summarises the data sources used in the evaluation of PPIE across the AI‐Multiply project. All data were drawn from existing project activities and documentation, with systematic analysis undertaken for the purpose of this study. Researcher interviews are planned as part of a future evaluation phase and are not included here.

### 3.1 CUBE Framework Analysis

**Quantitative data** were analysed using descriptive statistics and plotted as crosshair diagrams. Circles towards the centre suggest a weaker voice and less influence; those near the edge indicate more positive experiences. Circle size reflects the number of contributors selecting that point.

**Qualitative data** (free‐text) were analysed thematically [35] and visualised using word maps. Each work package had a finalised CUBE report summarising findings and tailored recommendations. These were circulated for feedback and to inform future practice.

### 3.2 Data Synthesis Approach

As our evaluation involved multiple methods that generated several data components, we opted to employ a ‘follow‐a‐thread’ approach to integrate key findings from across each. This approach is commonly used in mixed and multi‐method studies to integrate findings and is most often employed in health services, public health, humanities and medical education research [36]. ‘Following a thread’ involves initial interrogation of each component to identify potentially key themes relevant to the research question. A promising theme is then followed across the other components for further support and explanation. This creates a multifaceted picture of the phenomenon of interest [37]. For example, when elucidating the theme of ‘*Movement was needed on both sides’*, the first point of interest was noted in a feedback communication relating to conducting the PiiAF. Here, researchers expressed concern that they did not have enough expertise to plan PPIE involvement and impact in such a comprehensive way. This led to a search for similar points in the impact log and CUBE link to apprehension, which then extended to finding similar points relating to our PPIE partners. This technique supports the values of participatory and co‐produced research by enabling diverse perspectives to be integrated across multiple sources of data.

## 4 Findings

We identified three overarching themes from across the data components, relating to the issues and challenges to facilitating PPIE in AI and big data health research. These were the following: *differing priorities*, *movement needed on both sides* and *the value of published guidance*. Descriptions of these themes and examples of data from across the evaluation components are presented below.

### 4.1 Differing Priorities: A Core Challenge in Aligning Public and Researcher Perspectives

One of the key challenges in delivering meaningful PPIE within AI‐Multiply was the initial misalignment between the priorities of PPIE partners and those of researchers. This divergence often reflected a deeper tension: public contributors focused on person‐centred outcomes grounded in lived experience, while researchers were guided by what was feasible within existing data infrastructure and service priorities. In the context of big data and AI research, where data are both a resource and a constraint, this disconnect presented a substantial barrier to collaborative goal setting.

It was clear from our impact logs that researchers and PPIE partners generally identified different priorities for the work packages. During the early stages of involvement, our PPIE partners were asked to consider what outcomes for people living with MLTCs it would be beneficial for AI to predict. PPIE partner priorities had a strong focus on individual experiences and outcomes, with suggestions that included quality of life, patient satisfaction, periods of wellness and frequency and speed of negative events. Contributors also raised concerns around the sensitivity of predicting outcomes such as mortality and the importance of considering the audience for any such predictions.

Patient perspectives focused on quality of life, periods of wellness and independence – none of which were in the data.

—Data Engineering Impact Log Observation

In contrast, outcomes identified by the research team included hospital readmission and the relevance of predicting death, metrics commonly valued in healthcare system design and evaluation. Such discrepancies were difficult to resolve because the types of data needed to address PPIE partner priorities were often not available within existing electronic health records.

[The work package is] more focused on what researchers wanted and what data was available.

—WP1 CUBE Survey—PPIE1

This tension was especially apparent in one work package focused on co‐designing trial emulation studies. Although contributors generated important and relevant ideas, progress was repeatedly hindered by data limitations.

It felt like we might be being quite negative as we are more focused on what isn't possible to do with the data. Lots of nice ideas from the participants are sadly not possible to do with the limitations in the data.

—Main impact log, Researcher WP5

Concerns also emerged about the lack of linked data between different providers (e.g., dentists, pharmacists and specialists), which further limited the scope of involvement. Contributors were often surprised to learn how fragmented or incomplete health records were, something they felt undermined both research utility and the realism of PPIE contributions.

Contributors were surprised at how fragmented the data was. Frustration that key aspects of lived experience were not represented.

—Data Engineering Impact Log

This theme illustrates a core challenge specific to AI and big data research: the constraints imposed by available datasets can directly undermine the alignment of research priorities with public perspectives. While tensions reduced over time, particularly as understanding of data realities improved, managing expectations and maintaining engagement amid such limitations required continual work by the PPIE team. The role of PPIE leadership in mediating these issues is explored in Theme 2.

To further illustrate how these differing priorities manifested across the AI‐Multiply project, we developed a summary table comparing the public and researcher priorities across each work package (see Table 2). This highlights recurring tensions, particularly those rooted in limitations of available data, and illustrates where alignment was eventually achieved or remained unresolved.

**Table 2:** Comparison of PPIE versus researcher priorities by work package.

Work package	PPIE partner priorities	Researcher priorities	Tensions or resolutions
WP1: Data preparation	Wanted full data linkage across care settings (e.g., pharmacy, dental and specialists). Focus on person‐centred journeys.	Focused on preparing and linking existing data from EHRs; constrained by what's available.	Contributors were surprised at how fragmented the data was. Frustration that key aspects of lived experience were not represented in the data WP1 CUBE report.
WP2: Predictive clustering	Emphasis on wellness, early deterioration, quality of life and avoiding predictive use of mortality.	Focused on clinical outcomes like hospital admissions and mortality, based on routinely collected data.	Clear mismatch between public priorities and what data allowed; contributors concerned their suggestions were too often unworkable WP2 CUBE report.
WP3: Epidemiology	Focused on understanding experience of comorbidities, medication side effects and more relatable outcomes. Prioritised practical relevance and clear communication.	Focused on identifying clusters and trajectories using EHR data.	Felt researcher‐driven; contributors called for clearer inclusion earlier in planning and more time to discuss complex concepts. Mixed views on voice and clarity; limited time and jargon raised concerns.
WP4: Interdisciplinary working (Ethnography and Mission Statement Development)	Valued inclusion in technical spaces and equitable team dynamics; wanted clear roles and expectations.	Observing how ideas and collaboration emerge across disciplines in practice.	Relatively aligned; some concern about confidence and contribution levels; generally positive perception of being heard.
WP5: Trial emulation and translation	Suggested functional outcomes (e.g., independence, fatigue) and real‐world trial questions.	Needed to stick to what was recorded in EHRs (e.g., prescriptions, readmissions).	Strongest point of tension; many public suggestions were not possible. **Traffic light system** was developed to manage expectations and transparently map feasibility.

### 4.2 Movement Was Needed on Both Sides: From Apprehension to Integration

This theme reflects the two‐way process of adaptation that took place as researchers and public contributors learned to work together within a technically complex project. At the outset, both groups expressed uncertainty: contributors questioned their ability to engage with unfamiliar methods like machine learning or clustering, while researchers voiced concerns about how to communicate complex ideas clearly and how public input could meaningfully inform technically constrained research.

To address this, the PPIE team delivered introductory workshops on AI and data science. These sessions were positively received and helped lay the groundwork for contributor engagement.

I really appreciated the introduction sessions about AI as my understanding of AI, machine learning and data collection was very limited.

—WP1 CUBE survey—PPIE5

Despite this support, contributors continued to vary in their confidence and familiarity, particularly in larger or more technical settings.

Hope I'll get better as I become more familiar.

—WP1 CUBE survey— PPIE10

Some of our PPIE members are very well informed on the topic ‐ I have been less so ‐ may have held back a bit in contributing.

—WP1 CUBE survey—PPIE7

On the researcher side, some work packages were initially hesitant to embed PPIE, particularly where the focus was highly technical. Researchers were unsure how to explain their work in accessible terms and questioned how relevant public input could be when data constraints limited design flexibility.

Sometimes it feels like we're bringing people in too early before we've really figured out what we're doing ourselves.

—ECR Feedback WP2

However, over time, attitudes shifted. Impact logs and feedback documents revealed a growing appreciation of PPIE's role and influence.

Attendance by the number of researchers in the meetings showed their openness to PPIE.

—WP2 CUBE, PPIE6

[WP2 researcher] put PPIE into their presentation for the Full Team Meeting and said they were going to get people involved with her paper!

—Main Impact Log, PPIE Management Reflection

A key driver of this shift was the deliberate integration of public contributors into technical spaces, including whiteboard sessions and data engineering meetings. Initially, some researchers resisted this approach, concerned that public participation would disrupt technical discussion. To address this, the PPIE lead handpicked two experienced contributors, one with AI knowledge, the other with data familiarity, to attend regularly.

Initially, the researchers were wary of adding PPIE to the data engineering meetings.

—Data Engineering Impact Log

Over time, researchers adapted, developing a system where each contributor attended alternate meetings and shared updates between them. This model proved sustainable and effective, and the contributors involved were later invited to comment on papers, co‐author outputs and shape grant development.

ECRs were another group where integration had a marked impact. Dedicated PPIE‐ECR sessions gave researchers the opportunity to present ideas informally, refine their thinking and gain real‐time feedback from contributors. These sessions also helped ECRs build confidence in communicating complex ideas clearly and helped contributors feel they were supporting the next generation of researchers.

Instead of feeling as though my research did not matter, the PPIE group emphasised the importance of my work.

—ECR Feedback, WP3

The PPIE group challenged me to think about alternative ways to implement my machine learning tools.

—ECR Feedback, WP2

Across these examples, both groups made compromises and grew. Contributors gained fluency and confidence in AI and big data methods; researchers became more open, responsive and reflective. While not all tensions disappeared, the movement on both sides resulted in a more integrated and collaborative model of PPIE than was initially expected. Contributors were increasingly seen as equal participants, even in discussions once considered too technical for involvement.

One needs to be on their toes at all times as anything goes really with the type of questions and comments they bring up.

—Impact Log, Researcher WP3

These reflections show that integration was not just procedural but relational, built over time through repeated interaction, reflection and trust.

### 4.3 Value of Published Guidance: Why the Basics Still Matter in Complex Projects

Although AI and big data research present unique challenges, many of the issues raised by contributors reflected long‐standing concerns about core PPIE practice. This theme highlights that difficulties encountered across work packages often stemmed not from the complexity of the research itself, but from inconsistent application of well‐established guidance on how to involve people meaningfully. Public partners frequently identified shortcomings in preparation, facilitation and follow‐up, areas central to frameworks such as the UK Standards for Public Involvement [5].

**Figure 1:**
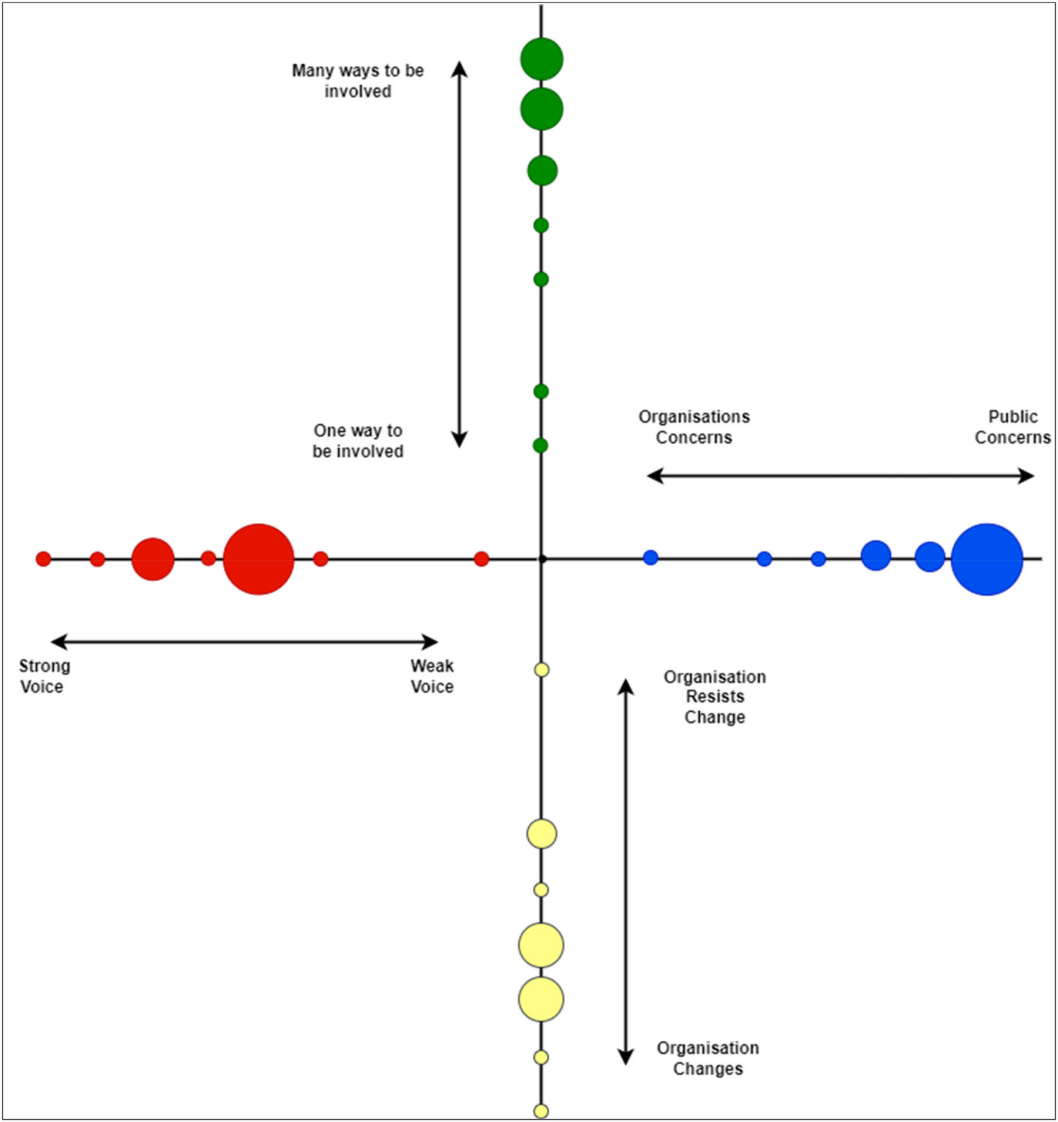
PPIE members' perceptions of their involvement within the mission statement development. Crosshair plot depicting PPIE members' perceptions of their involvement within mission statement development. The size of the circles indicates the number of members selecting that value on the scale.

When responding to our CUBE surveys, contributors largely perceived themselves as having a strong voice, many ways to be involved and good alignment with research concerns (example shown in Figure 1). In the CUBE plot, each circle represents a contributor's perception of their involvement, with its position reflecting scores on four dimensions. Larger circles indicate multiple contributors selecting the same value. Circles near the edge suggest stronger alignment and more positive experiences; those closer to the centre reflect concerns or lower perceived inclusion.

However, corresponding free‐text responses revealed more nuanced concerns, especially around preparation, follow‐through and feedback, underscoring that numerical scores can mask subtle but important issues in session quality.

It would be useful if suggestions not incorporated were identified with an explanation… we learn from these.

—WP2 CUBE, PPIE6

Sometimes it feels a little rushed – 60 minutes.

—WP3 CUBE, PPIE3

These concerns mirror established guidance on clarity of purpose, preparation, discussion time and feedback, suggesting that core PPIE standards remain central, even in technically demanding research.

This was further illustrated in the CUBE evaluations of the mission statement session. Contributors appreciated the open format and opportunities for involvement, but some still felt constrained by time, dominant voices or unclear outcomes. Suggestions for more inclusive routes to participation, such as chat, email or anonymous input, underscore the need for facilitation methods that accommodate diverse communication styles and comfort levels.

I had a lady who was speaking too much so I could not get involved much.

—Mission Statement CUBE, PPIE2

We also observed that even when contributors had many ways to be involved, the lack of feedback loops or unclear expectations sometimes led to uncertainty about impact.

I felt they were willing to listen… but unsure if it's implemented.

—WP1 CUBE, PPIE23

These are basic issues, but in a large, interdisciplinary project with multiple work packages, ensuring consistent delivery of these principles proved difficult.

The importance of experienced PPIE leadership in navigating and enforcing these standards became increasingly apparent. For example, the decision to embed contributors in researcher meetings or to adjust agendas so PPIE came earlier were practical applications of guidance on inclusion and influence, but they required leadership and negotiation to implement. The leadership role was especially crucial in managing relationships and creating continuity across work packages.

In summary, this theme shows that the value of published PPIE guidance remains strong even in AI and big data research. What is often lacking is not guidance, but the capacity, planning and leadership needed to put it into practice. Our evaluation confirms that consistent attention to these ‘basics’, such as preparation, inclusion, clarity and feedback, is essential for meaningful involvement, regardless of the technical complexity of the research.

## 5 Discussion

In this paper, we explored the issues and challenges of facilitating PPIE in AI and big data health research, using AI‐Multiply, a large, multidisciplinary, multi‐method project, as an exemplar. Our integrated analysis identified considerations for incorporating PPIE into similar projects. Diverging priorities between researchers and PPIE partners initially created a divide, exacerbated by data limitations. Bridging this gap required mutual adaptation. Practical PPIE principles from established guidance were essential in achieving progress.

### 5.1 Bridge the Divide

Tensions between researchers and PPIE partners are well‐documented. Researchers unfamiliar with PPIE or working in technical fields may lack confidence or question the relevance of involvement. Some are unsure how to share control or integrate public perspectives and may view PPIE as resource‐intensive for limited gain [38, 39]. As recent surveys have shown, researchers want practical guidance and case studies to increase confidence in PPIE, especially in technical contexts [21].

In AI‐Multiply, exposure to PPIE itself led to attitude shifts. As both ECR and Senior researchers saw the value of contributor perspectives and co‐production dynamics, they became more willing to engage. This suggests that expanding direct experience of PPIE may be as important as formal training.

Apprehension also existed among public contributors. Some felt they lacked sufficient expertise in AI or large‐scale research to contribute meaningfully. This mirrors previous findings showing that limited understanding of technical concepts can restrict involvement [40]. The need for baseline understanding is also recognised in commercial AI development [28]. Our project's introductory training helped build confidence and establish a foundation for engagement.

Beyond training, our approach to integration played a key role in bridging divides. Proactive, flexible PPIE leadership was vital. By embedding contributors into technical meetings and encouraging participation in research outputs, we demonstrated the feasibility, and value, of involving the public in areas often seen as off‐limits. This required experienced coordination, and our findings suggest this level of integration is novel in such a complex, multi‐component context.

While the value of PPIE coordinators is well established [21, 38], our approach demonstrates the added benefit of strategic, persistent leadership to embed contributors throughout the research lifecycle. We recommend this approach for similar projects.

### 5.2 Manage Expectations of Available Data

Even as priorities aligned, practical constraints limited how responsive researchers could be. Public contributors offered many valuable ideas that could not be pursued due to missing data in health records. This recurring tension, between public priorities and data availability, does not appear widely documented in the PPIE literature but emerged as a major theme in our study.

Improving EHR data quality is beyond this paper's scope, but addressing gaps could enhance research relevance. Prior work suggests improving existing data quality [41] and using patient‐reported outcomes tools like those tested in neuroendocrine cancer care [42]. Contributors consistently highlighted that quality of life and functional status, rarely recorded in EHRs, were highly important to patients.

In the meantime, projects should manage expectations clearly. Contributors must understand the limits of the data and what is achievable. Open dialogue around data constraints can reduce frustration and build trust. The development of the traffic light system to indicate the feasibility of suggested research ideas within AI‐Multiply is an example of how we managed expectations and encouraged discussion.

### 5.3 Get the Basics Right

Most feedback from contributors concerned well‐known principles: allowing enough time, sharing materials in advance and providing clear feedback. These align with the UK Standards and broader guidance [5, 43]. That these principles were still inconsistently applied suggests ongoing barriers to implementation.

Among AI researchers, limited awareness of PPIE guidance may be one barrier. PPIE is often not seen as a skill requiring training, yet it involves advanced communication and facilitation, especially in complex domains. Enthusiasm alone is not enough; good PPIE requires preparation and people skills.

Incorporating PPIE skills development into the training of ECRs may also raise awareness of these principles. A need for PPIE training early in a research career has been noted previously in a report on the progress of public involvement within NIHR‐funded research [44]. Exposing ECRs to PPIE at early stages has been previously acknowledged as a strategy that can build research cultures sensitive to the potential contribution of PPIE and help to develop expertise that is needed to avoid tokenistic involvement [16]. In AI‐Multiply, our ECRs appreciated opportunities and the benefits of dedicated sessions to directly interact with our PPIE partners in a non‐threatening forum. Offering similar sessions in other large research projects may help meet this need for skills development.

Experienced leadership can help implement basic standards, but building a supportive research culture is also essential. Introducing PPIE training early in research careers may improve awareness and capability. This echoes findings from NIHR reports and other evaluations [16, 44].

In our project, dedicated ECR–PPIE sessions created a safe space for dialogue. These were well received by both groups and may help address gaps in training. Similar initiatives could support capacity‐building in other large research programmes.

### 5.4 Strengths and Limitations

A key strength of this study was the active involvement of public contributors throughout the evaluation process. Contributors shaped the design of evaluation methods, including input into the use of the CUBE framework, and were involved in interpreting data and drafting the manuscript. One public contributor is a co‐author, and others provided detailed feedback on early drafts. This helped ground the evaluation in public priorities and enhanced the clarity and accessibility of reporting.

While our findings offer novel insights and suggestions for PPIE in AI and big data research, some limitations must be acknowledged. Our reflections were based on text‐based feedback in impact logs, along with anonymous quantitative and free‐text data from CUBE surveys. Anonymity likely encouraged honest responses, but one‐to‐one interviews with researchers and contributors may have yielded more detailed insights and allowed follow‐up.

CUBE survey response rates varied, with as few as eight contributors responding at times. Some public partners reduced involvement or left the project, meaning not all views are represented. Contributors also reported that CUBE question wording was occasionally unclear, which may have limited participation. Following this feedback, we redrafted the questions into a plain English format, which was well received. Adapting the language to fit the project and public audiences may improve clarity.

The evaluation methods that we have employed may have resulted in a bias towards PPIE contributor input and a paucity of researcher perspectives. The CUBE is aimed at gathering the views of PPIE partners, and an equivalent for the research team should be employed. We plan one‐to‐one interviews with researchers from each work package in AI‐Multiply to understand their views on PPIE conduct and impact.

Our evaluation focused primarily on the ‘Newcastle’ PPIE group. These contributors were already involved in PPIE across the AI‐Multiply project, and most were experienced in research involvement. We did not collect formal demographic data, which limits our ability to assess representativeness. As such, our findings may not reflect the views of contributors from other regional or demographic backgrounds, including the London group.

## 6 Implications for Future Practice

Our findings will support the development of practical tools for PPIE in complex, data‐driven health research. After completing further evaluation, including researcher interviews, we aim to co‐produce planning resources that address key challenges: managing expectations around data limitations, embedding contributors in technical spaces and applying PPIE standards consistently across interdisciplinary teams.

## 7 Conclusion

Our reflections on PPIE in AI‐Multiply highlight both challenges specific to AI and big data, and those familiar from broader PPIE practice. Consistent application of existing guidance emerged as one of the most effective strategies. Our experience underscores the effort and time required for meaningful involvement, and the importance of experienced, proactive leadership. By building shared understanding and applying established standards, future projects may be better equipped to integrate public perspectives, though whether this improves research impact remains an open question.

## Author Contributions

**Alexandra Thompson:** conceptualisation, data collection, analysis, writing – original draft preparation, writing – review and editing. **Barbara Hanratty:** conceptualisation, writing – review and editing. **Elizabeth A. Remfry:** writing – review and editing. **Duncan J. Reynolds:** writing – review and editing. **Victoria Bartle:** writing – review and editing. **Michael R. Barnes:** writing – review and editing. **Nick J. Reynolds:** writing – review and editing.

## Ethics Statement

This study received ethical approval from Newcastle University Research Ethics Committee (Ref. 49847/2023).

## Conflicts of Interest

The authors declare no conflicts of interest.

## Data Availability

The data that support the findings of this study are available on request from the corresponding author. The data are not publicly available due to privacy or ethical restrictions.
